# CRISPR Screen Reveals PACT as a Pro-Viral Factor for Dengue Viral Replication

**DOI:** 10.3390/v16050725

**Published:** 2024-05-03

**Authors:** Shwetha Shivaprasad, Wenjie Qiao, Kuo-Feng Weng, Pavithra Umashankar, Jan E. Carette, Peter Sarnow

**Affiliations:** 1 Department of Microbiology & Immunology, Stanford University SOM, Stanford, CA 94305, USA; qwenjie@stanford.edu (W.Q.); arette@stanford.edu (J.E.C.); psarnow@stanford.edu (P.S.); 2 Molecular Biology and Genetics Unit, Jawaharlal Nehru Centre for Advanced Scientific Research, Bengaluru 560064, Karnataka, India; pavithraus@jncasr.ac.in

**Keywords:** dengue virus, CRISPR screen, host–virus interactions

## Abstract

The dengue virus is a single-stranded, positive-sense RNA virus that infects ~400 million people worldwide. Currently, there are no approved antivirals available. CRISPR-based screening methods have greatly accelerated the discovery of host factors that are essential for DENV infection and that can be targeted in host-directed antiviral interventions. In the present study, we performed a focused CRISPR (Clustered Regularly Interspaced Palindromic Repeats) library screen to discover the key host factors that are essential for DENV infection in human Huh7 cells and identified the Protein Activator of Interferon-Induced Protein Kinase (PACT) as a novel pro-viral factor for DENV. PACT is a double-stranded RNA-binding protein generally known to activate antiviral responses in virus-infected cells and block viral replication. However, in our studies, we observed that PACT plays a pro-viral role in DENV infection and specifically promotes viral RNA replication. Knockout of PACT resulted in a significant decrease in DENV RNA and protein abundances in infected cells, which was rescued upon ectopic expression of full-length PACT. An analysis of global gene expression changes indicated that several ER-associated pro-viral genes such as ERN1, DDIT3, HERPUD1, and EIF2AK3 are not upregulated in DENV-infected PACT knockout cells as compared to infected wildtype cells. Thus, our study demonstrates a novel role for PACT in promoting DENV replication, possibly through modulating the expression of ER-associated pro-viral genes.

## 1. Introduction

The dengue virus is a mosquito-borne RNA virus that infects ~100–400 million people globally every year [1]. Currently, there are no approved antiviral treatments available for DENV infection. While direct-acting antivirals targeting DENV proteins have shown promise in pre-clinical trials, complementary host-directed therapies are also being evaluated to overcome the emergence of drug resistance and for broad spectrum activity against multiple DENV serotypes [2].

DENV is an enveloped, single-stranded, positive-sense RNA virus belonging to the *Flaviviridae* family of viruses. The DENV genome encodes three structural and seven non-structural proteins that are required in distinct steps of the viral life cycle, including entry, translation, replication, assembly, and release [3]. The interactions of viral RNA and viral proteins with host cellular factors play a critical role in each of these steps, allowing the virus to multiply and disseminate. Functional genomics and proteomics-based tools have significantly accelerated the discovery of host factors that are essential for viral infection. CRISPR-Cas genetic screens and comprehensive identification of RNA-binding proteins by mass spectrometry (ChIRP-MS) have identified numerous host proteins that are essential for DENV infection. Some notable examples include proteins that function in the oligosaccharyl transferase (OST) complex, the ER membrane protein complex (EMC), the translocon-associated protein (TRAP) complex, and the dolichol-phosphate mannose synthase (DPMS) complex [4,5,6,7]. Many of these host proteins are also co-opted by other members of the *Flaviviridae* family, such as the Zika virus (ZIKV), West Nile virus (WNV), and yellow fever virus (YFV), to facilitate efficient replication. Occasionally, host factor requirements are conserved across different families of viruses due to similarities in their infection and immune evasion strategies. For example, TMEM41B is an integral endoplasmic reticulum membrane protein recruited by flaviviruses (DENV, YFV, and ZIKV) as well as coronaviruses (SARS-CoV-2) for remodeling ER membranes into viral replication factories [8,9]. Similarly, the ribosomal scaffold protein RACK1 was shown to alter the morphology of viral replication factories and promote viral RNA synthesis in both flavivirus- and COVID-19-infected cells [10].

Given the interest in identifying common host targets for the development of broad-spectrum antivirals, we performed a DENV screen using a focused CRISPR library targeting the SARS-CoV-2 RNA interactome identified through ChIRP-MS [11]. This sub-library has previously been used to screen for host factors involved in the infection of multiple RNA viruses, including other coronaviruses, encephalomyocarditis virus (EMCV), and influenza virus (IAV). In addition to the advantages of enhanced coverage and higher resolution provided by a smaller library, this approach allows us to leverage the knowledge gained from studying host–virus interactions of SARS-CoV-2 to identify novel host factors that are important for DENV infection [12]. In our study, we identified the Protein Activator of Interferon-Induced Protein Kinase (PACT/PRKRA) as a novel host factor that is co-opted by DENV and further explored its role in modulating DENV replication.

## 2. Materials and Methods

### 2.1. DENV CRISPR Mini-Library Screen

Ten million cells were used per screen to constitute about 1000× g RNA coverage. The library was infected with Hap1-adapted DENV2 [13] at an MOI of 2. The selected cells were collected 5 days after infection. The unselected starting population was used as an uninfected reference. gDNA was isolated from both selected and uninfected control cells, and the inserted guide RNA sequences were amplified from the gDNA by flanking primers and prepared for next-generation sequencing. The sequencing data were analyzed with the MAGeCK algorithm.

### 2.2. Plasmids

DENV-2 infectious clone 16681 was a generous gift from Dr. Karla Kirkegaard, Stanford University. HAP1-adapted DENV-2 was derived from infectious clone 16681 [13]. The pDENV2-Luc infectious clone and the DENV replicon-containing plasmids were generated as described before by Dr. Jan Carette’s lab at Stanford University [6]. pLentiCRISPR V2 (Addgene, #52961), pLentiCRISPR V2 mCherry, pLenti c-MYC-DDK-Puro GFP (Addgene, #123299), pLenti-C-Myc-DDK-IRES-Puro-PRKRA (Addgene, #106113), PRKRA-delta1 (Addgene, #106114) and PRKRA-delta23 (Addgene, #106117), were obtained from Addgene.

### 2.3. Cell Culture

Huh7 wildtype and PACT knockout cell lines were cultured as monolayers in Dulbecco’s modified Eagle’s medium supplemented with 10% fetal bovine serum, 100 units of penicillin/mL, 100 µg of streptomycin/mL, 1× NEAA (non-essential amino acid medium), and 2 mM L-Glutamine (Gibco/Life Technologies, Carlsbad, CA, USA). Dr. Jillian Lund from the Sarnow lab is acknowledged for generating the clonal PACT knockout cell line. The HEK293/FH-PACT cell line was a kind gift from Dr. Thomas Tuschl at Rockefeller University, New York, USA [14]. These cells were cultured in DMEM, additionally supplemented with 15 µg/mL Blasticidin and 100 µg/mL Hygromycin. PACT expression was maintained by adding doxycycline at a final concentration of 1 ng/µL. All cell lines were grown at 37 °C with 5% CO_2_.

### 2.4. Cloning and Knockout Generation

PACT knockout cell lines were generated by cloning 20 bp guide RNA sequences targeting PACT into pLentiCRISPRV2 or pX458 plasmids. HEK293FT cells were transfected with pLentiCRISPRV2 plasmids expressing sgRNAs #1, #2, or #3 against PACT to generate the respective lentiviruses. The sequences of the guide RNAs are

ATTCAGGTATTACACGAATA (sgRNA#1), GGCGAAACATAGAGCTGCAG (sgRNA#2), and AATTGGCTATTCATCATGGC (sgRNA#3). Huh7 cells were transduced with these lentiviruses, and PACT knockout cells were selected by puromycin treatment. One of the guide RNAs, sgRNA #3, was also cloned in the Cas9-expressing pX458 plasmid. Huh7 cells transfected with pX458-sgPACT #3 were single-cell sorted based on GFP into 96-well plates using a BD Influx cell sorter at the Stanford Shared FACS facility. Clonal cell lines were allowed to expand from a single cell, and genomic DNA was isolated for sequencing-based genotyping of targeted alleles. For this, a 300–500 base-pair (bp) region that encompassed the guide RNA-targeted site was amplified, and the sequence of the PCR product was determined by Sanger sequencing. Subclones were chosen where all alleles were mutated with insertions or deletions that were not a factor of 3. Knock-out subclones verified by genotyping were further confirmed by Western blot using antibodies against PACT.

### 2.5. Lentiviral Transduction

5 × 10^6^ HEK293 FT cells were co-transfected with pCMV-dR8.2 dVPR (1.3 µg), pCMV-VSV-G (0.86 µg), pAdVantage (0.55 µg), and pLenti-GFP/PACT/sgRNA encoding plasmids (2.2 µg) using 15 µL of TransIT-LT1 transfection reagent (Mirus #MIR 2300) in 150 µL optiMEM. The transfection mix was incubated for 30 min and then added to the cells. Supernatants containing lentiviruses were harvested at 48 h and 72 h post-transfection, filtered using a 0.45 µm filter, and stored at −80 °C for further use. For gene silencing or overexpression, cells were transduced with the relevant lentivirus at an MOI  ≥  2 for 6 h followed by the addition of a fresh medium. Cells stably expressing the gene of interest were selected using puromycin (InvivoGen, San Diego, CA, USA) at a concentration of 5 µg/mL for Huh7 cells and 1 µg/mL for HEK293/FH-PACT cells, along with untransduced cells as negative controls. 

### 2.6. Virus Infections

Infectious DENV cDNA clones were digested with XbaI and in vitro transcribed to generate capped DENV RNA, as described below, followed by transfection into BHK21 cells in 24-well plates. Supernatants were collected 48 h post-transfection and used to infect C6/36 cells overnight in a T75 flask containing 3 mL of complete medium. A total of 15 mL of complete medium was added the following day, and a virus supernatant was collected 6 days post-infection. Virus titers were determined using standard plaque assays on BHK21 cells. Virus infections were carried out for 1.5 h in 2% FBS-containing medium at the desired multiplicity of infection (MOI). MOI refers to the number of plaque-forming units used per cell. DENV-1 (276RKI, BEI NR-3782), DENV-3 (VN/BID-V1009/2006, BEI NR-44088), DENV-4 (BC287/97, BEI NR3806), and ZIKV Puerto Rico strain (PRVABC59, BEI NR-50240) were obtained from BEI resources, propagated in C6/36 cells, and titered by plaque assay on Huh7.5.1 cells. West Nile Virus (Kunjin strain CH 16532) was a gift from Dr. J. F. Anderson. The yellow fever virus was generated by culturing the yellow fever YF-VAX 17D-204 vaccine in HAP1 cells. Chikungunya virus (CHIKV 181/25) was a gift from Dr. Margaret Kielian. All virus infection assays were conducted following the BSL2 biosafety protocol.

### 2.7. In Vitro RNA Transcription

The DENV-2 16681 infectious cDNA clone and the DENV2 16681 replicon containing plasmids were linearized with XbaI, and in vitro transcriptions were performed using the MEGAscript T7 transcription kit (AM1334). 5 µg of the XbaI-linearized plasmid was incubated with 1.3 µL of 75 mM rATP, 6.7 µL each of 75 mM rCTP, rGTP, and rUTP, 10 µL of 10 X reaction buffer, 10 µL of T7 enzyme mix, and 12.5 µL of 5′GpppA cap analog (S1406S, NEB) in a final reaction volume of 100 µL for 30 min at 30 °C. 2.6 µL of 75 mM rATP was added to the reaction and further incubated for 4 h at 30 °C. The reactions were treated with DNAse, and RNA was purified using the RNEasy mini kit according to the manufacturer’s protocol.

### 2.8. Transfection and Electroporation

For DENV2 virus generation, 1.5 × 10^5^ BHK21 cells were transfected with 1 µg of DENV2 RNA using Lipofectamine 3000 in a 24-well plate. The medium was changed 2 h post-transfection. For replicon assays, 2 × 10^6^ Huh7 cells were washed twice with 10 mL 1X PBS, once with 2 ml of BioRad (Hercules, CA, USA) electroporation buffer (Catalog number #1652677), and then resuspended in 100 µL of cold electroporation buffer. A total of 4 µg of DENV-RLuc replicon RNA and 1 µg of Fluc mRNA (Trilink Biotechnologies (San Diego, CA, USA) L-7602) were added to cells, and the mix was transferred to a 1 mm electroporation cuvette (BioRad #1652083). Cells were electroporated at 120 V, with 10 pulses each with a pulse length of 1.5 ms and a pulse interval of 1.5 s in a BioRad gene pulser XCell electroporation system. Following electroporation, the cells were immediately resuspended in an antibiotic-free DMEM medium and seeded in 96-well plates. They were harvested in 1× passive lysis buffer at the indicated time points for measuring luciferase expression.

### 2.9. Luciferase Assay

For measuring Renilla luciferase activity, cells were washed once with PBS and harvested in 100 µL of 1× passive lysis buffer (Promega, Madison, WI, USA) for each well of a 96-well plate. A total of 10 μL aliquots of the lysate were mixed with 50 µL of Renilla Glo luciferase assay reagent (Promega) in white opaque plates, and luminescence was measured in the Glomax 20/20 luminometer with a 10 s integration time. For dual luciferase assays, cells were harvested as above at the indicated time points, and luciferase activity was measured using the Dual Luciferase Reporter Assay system (Promega), according to the manufacturer’s instructions.

### 2.10. Western Blot

Cells were washed with PBS, lysed in an RIPA buffer, and resolved on a 10% SDS-polyacrylamide gel. Proteins were transferred onto a PVDF membrane (Millipore), blocked with 5% non-fat milk in PBS-T, and the membranes were incubated with primary antibodies. Horse radish peroxidase-conjugated secondary antibodies were used to visualize proteins using Pierce ECL Western Blot Substrate (Thermo Fisher Scientific, Waltham, MA, USA) following the manufacturer’s protocol. The following primary antibodies were used for western blot analysis: Anti-NS3 antibody (GTX124252, GeneTex), Anti-NS4B antibody (GTX124250, GeneTex), Anti-Actin antibody (A2066, Sigma), Anti-PACT antibody ((D9N6J, Rabbit mAb #13490), and Anti-GAPDH antibody (GTX627408, GeneTex).

### 2.11. qPCR

Cells were harvested in TRIzol (Invitrogen), and the total RNA was isolated according to the manufacturer’s instructions. A total of 1 µg of RNA was used for cDNA synthesis using the High Capacity RNA-to-cDNA kit (Thermo Fisher, 4387406). A total of 2 μL of cDNA was used to amplify the target genes using the Power Up SyBR Green master mix (Thermo Fisher, A25742). Ct values of target genes were normalized to Ct values of the housekeeping gene, GAPDH, to calculate fold changes in RNA abundances.

## 3. Results

### 3.1. Focused CRISPR Screen Identifies Host Factors Important for DENV Infection

**Figure 1:**
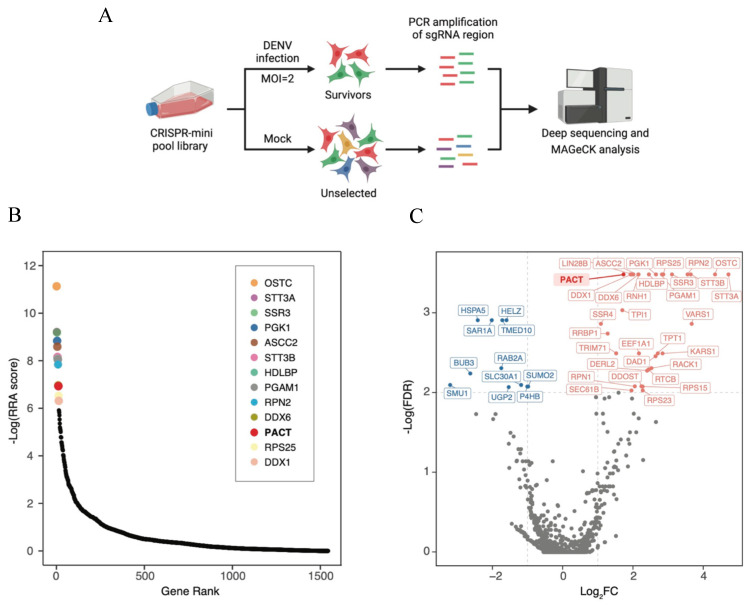
Bubble plot of results from the DENV CRISPR mini-pool library screen. (**A**) Schematic for the CRIPSR mini-pool library screen in Huh7 cells. (**B**) Candidate genes identified by the CRISPR-Cas9 screen. Data analysis was performed using the MAGeCK algorithm to identify enriched sgRNA, and the genes were ranked by robust rank aggregation (RRA) scores. (**C**) Volcano plot displaying the log2 fold change and FDR for genes identified in the screen. Genes with a threshold of FDR < 0.01 and log2 fold change > ±1 are labeled.

To investigate whether SARS-CoV-2 RNA-binding proteins have functional roles in DENV infection, we used a focused CRISPR mini pool library targeting 1331 proteins from the SARS-CoV-2 interactome for a live–dead screen with DENV infection [11]. The library was challenged with DENV2 at an MOI of 2 for 5 days, cells that survived were collected, and sgRNAs were PCR-amplified and analyzed by next-generation sequencing (Figure 1A). MAGeCK analysis of DENV-infected samples in comparison with the unselected starting population identified a set of enriched genes (Figure 1B). Using a significance threshold of FDR<0.01, we identified 32 proviral factors and 11 antiviral factors associated with dengue infection (Figure 1C, Supplementary Table S1). These include extensively studied proviral factors for dengue replication, such as the OST complex (RPN2, OSTC, STT3A, and STT3B), RRBP1, HDLBP, RPS25, and DDX6 [7,15], along with previously recognized antiviral factors for YFV, HSPA5, and SMU1 [16]. The cellular protein PACT stood out as one of the top-ranking hits on our screen, suggesting that it could play a pro-viral role in DENV infection. This was a curious finding because we have recently discovered that the mosquito ortholog of PACT, loquacious, has pro-viral activities in DENV-infected mosquito cells [17].

### 3.2. PACT Is a Pro-Viral Factor for DENV Replication

**Figure 2:**
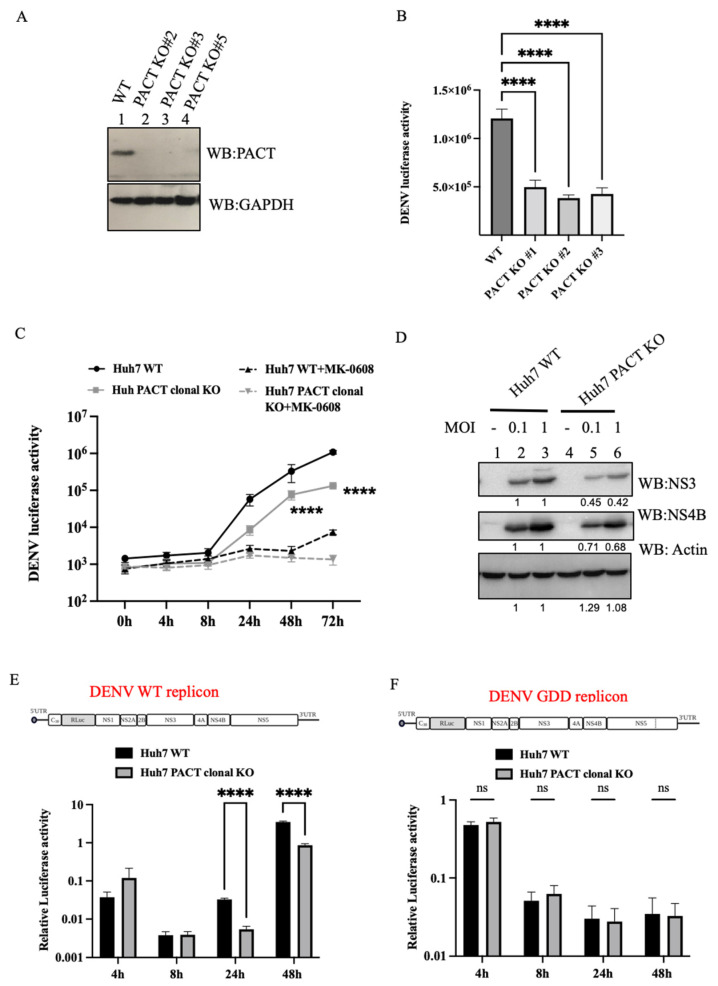
Effect of PACT knockout on DENV replication. (**A**) Western blot depicting depletion of PACT in Huh7 cells transduced with lentiviruses expressing sgRNA#1, sgRNA#2, or sgRNA#3 targeting the PACT gene. (**B**) Huh7 wildtype and PACT knockout cells were infected with DENV2-Luc reporter virus at an MOI of 0.1, and cells were harvested 48 h post-infection for measurement of luciferase activity. Luciferase expression in cell lysates is plotted as an average from three independent experiments (n = 3, **** *p* < 0.0001). (**C**) A clonal PACT knockout Huh7 cell line expressing sgRNA#3 was generated by cell sorting and infected with DENV2-Luc reporter virus at an MOI of 0.1. Infected cells were either untreated or treated with the viral replication inhibitor MK-0608. Luciferase expression was measured at the indicated time points and plotted as an average from three independent experiments (n = 3, **** *p* < 0.0001). (**D**) Western blot for DENV NS3 and DENV NS4B in Huh7 wildtypes and PACT knockout cells infected with DENV2 16681 at the indicated MOI. Densitometry values are indicated below the respective lanes. (**E**) Huh7 wildtype and PACT knockout cells were transfected with either DENV-RLuc wildtype replicon RNA or (**F**) DENV-RLuc replication defective (NS5-GDD) RNA together with a Firefly luciferase (FLuc)-expressing control mRNA. Cells were harvested at the indicated time points in six independent replicates, and relative luciferase expression is plotted as the ratio of Renilla and Firefly luciferase activities for both wildtype and replication-defective DENV RNAs (**** *p* < 0.0001).

To investigate the role of PACT in a DENV infection, we generated PACT knockout Huh7 cell lines through lentiviral transduction of three different guide RNA sequences (#1, #2, and #3) against PACT. Successful knockout of PACT was confirmed by Western blot analysis (Figure 2A). Both wildtype and PACT knockout cells were infected with Dengue luciferase-reporter virus (DENV-Luc), where the luciferase activity directly correlates with viral RNA translation and replication. At 48 h post-infection, luciferase activity was measured, and a 6–8 fold decrease in luciferase expression was noticed in PACT knockout cells compared to wildtype Huh7 cells. All three sgRNAs demonstrated similar effects on the luciferase activity, excluding potential off-target effects (Figure 2B). To further affirm the role of PACT in DENV infection, we generated a clonal PACT knockout Huh7 cell line expressing sgRNA#3 through single-cell sorting. Subsequent infection with DENV-Luc confirmed a sustained 6–8 fold reduction in DENV luciferase activity over time and significantly lower abundances of DENV NS3 and NS4B proteins in the PACT clonal knockouts relative to wildtype cells (Figure 2C,D), with no substantial effects on cell viability (Figure S1). We also compared DENV luciferase activities in wildtype and PACT knockout cells treated with 50 µM MK0608, a DENV RNA replication inhibitor [18] (Figure 2C). We observed no significant changes in the luciferase activity in PACT knockout cells treated with MK-0608 relative to the treated wildtype cells at the initial time points, suggesting that PACT does not affect initial viral RNA translation but has an impact on subsequent viral replication.

To further determine the stage of the viral life cycle at which PACT acts, we evaluated the effects of PACT depletion on DENV replication using DENV-luciferase expressing replicon RNAs. Wildtype and PACT knockout cells were transfected with replication-competent or replication-defective viral RNAs to distinguish between effects on viral RNA replication and translation. A firefly-luciferase expressing control RNA was co-transfected to normalize for any effects on global translation, cell viability, and metabolic activity. Relative luciferase activity was measured in transfected cells at different time points. At 48 h post-transfection, we observed a significant reduction in relative luciferase activity in PACT knockout cells transfected with replication-competent DENV RNA as compared to wildtype cells (Figure 2E). Conversely, no significant changes were noted with the replication-defective DENV RNA (Figure 2F). These results indicate that PACT affects viral RNA replication rather than viral RNA translation.

### 3.3. PACT Promotes Replication of DENV but Not Other Flaviviruses

**Figure 3:**
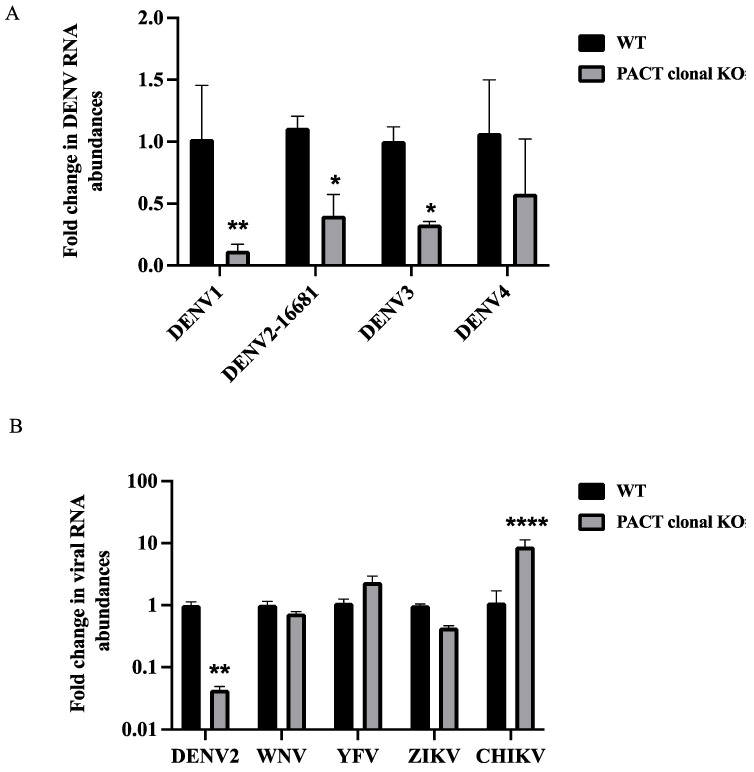
Effect of PACT knockout on distinct viral infections. (**A**) Huh7 wildtype and PACT knockout cells were infected with DENV-1, DENV-2, DENV-3, or DENV-4 at an MOI of 0.1, and viral RNA abundances were measured by qPCR using universal DENV primers. Data are represented as the average fold change over wildtype cells from three independent experiments (** *p* < 0.01, * *p* < 0.1). (**B**) Huh7 wildtype and PACT knockout cells were infected with Dengue virus (DENV), West Nile virus (WNV), yellow fever virus (YFV), Zika virus (ZIKV), or chikungunya virus (CHIKV) at an MOI of 0.1 and harvested 48 h post-infection. Viral RNA abundances were measured by qPCR using specific primers. Data are represented as average fold-change over wildtype cells (**** *p* < 0.0001, ** *p* < 0.01).

The importance of PACT in DENV infection was further validated by measuring viral RNA levels through quantitative PCR (qPCR) in wildtype and PACT knockout cells following virus infection. A significant reduction in viral RNA abundance was observed with three DENV serotypes, DENV1, DENV2, and DENV3 (Figure 3A). The impact of PACT depletion on other flaviviruses, including West Nile virus, yellow fever virus, and Zika virus, as well as chikungunya virus, which belongs to the *Togaviridae* family, was also investigated. Notably, no antiviral effects were observed in PACT knockout cells for these tested viruses (Figure 3B).

### 3.4. PACT Addback Rescues DENV Replication

**Figure 4:**
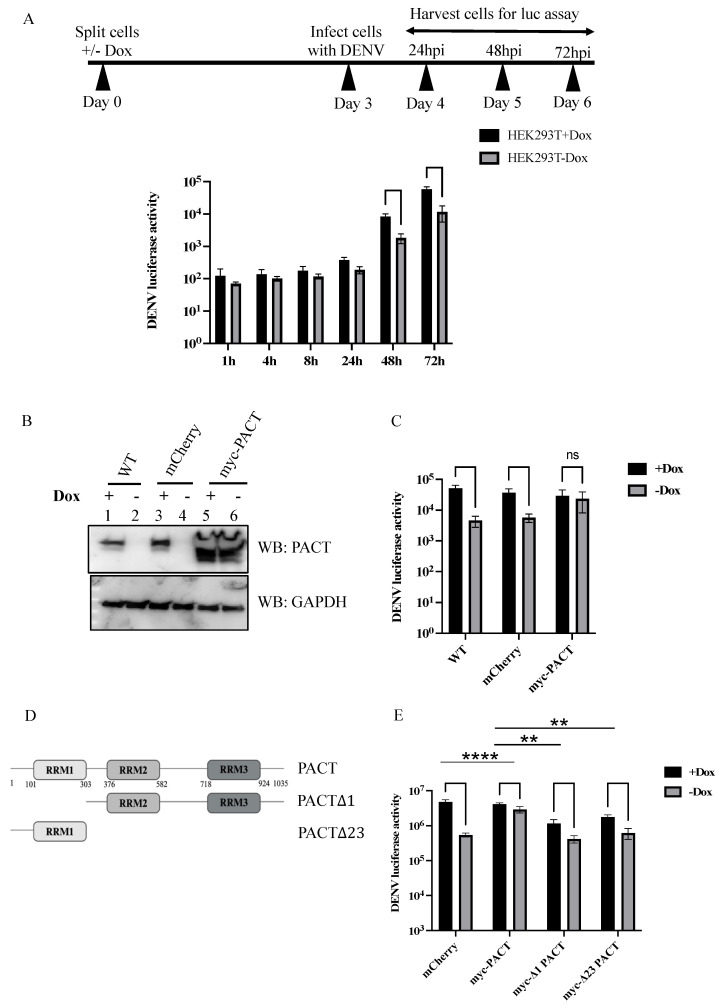
Effect of ectopic expression of PACT on DENV infection in HEK293/FH-PACT cells. (**A**) HEK293/FH-PACT cells were cultured in the presence or absence of doxycycline and infected with the DENV2-Luc reporter virus at an MOI of 0.1. Luciferase expression was measured from cells harvested at the indicated time points post-infection and plotted as an average of six independent replicates (n = 3, **** *p* < 0.0001). (**B**) Western blot for PACT protein expression and (**C**) luciferase expression from DENV2-Luc reporter virus-infected wildtype, mCherry knock-in, and myc-PACT knock-in HEK293/FH-PACT cells, cultured in the presence or absence of doxycycline (**** *p* < 0.0001). (**D**) Domain organization of PACT and its RRM deletion mutants. (**E**) HEK293/FH-PACT cells expressing wildtype or truncated versions of PACT were cultured in the presence or absence of doxycycline and infected with the DENV2 luciferase reporter virus at an MOI of 0.1. Luciferase expression was measured 72 h post-infection and plotted as an average of six independent replicates (**** *p* < 0.0001, ** *p* < 0.01).

Our attempt to rescue DENV replication in PACT knockout Huh7 cells through the ectopic expression of PACT failed due to cell death induced by PACT expression. We then employed a doxycycline-inducible HEK293/FH-PACT cell line in which endogenous PACT was knocked out but an FLAG/HA-tagged PACT (FH-PACT) was expressed upon doxycycline (Dox) induction [14]. Unlike Huh7 cells, no cytotoxicity was observed in the HEK293/FH-PACT cell line in the presence of Dox, i.e., FH-PACT expression. HEK293/FH-PACT cells treated with or without Dox were infected with DENV-Luc. We observed a significant decrease in DENV luciferase activity in cells without Dox, similar to that observed with PACT knockout Huh7 cells (Figure 4A). To test whether the phenotype could be rescued by PACT expression, we generated HEK293/FH-PACT cell lines stably expressing myc-PACT or a control mCherry protein (Figure 4B). DENV luciferase activity was successfully restored in cells expressing myc-PACT, comparable to those treated with Dox (Figure 4C). PACT contains three RNA recognition motifs (RRMs) that are involved in double-stranded RNA binding, protein–protein interactions, and activation of downstream kinases. In order to test the contribution of these RRMs to DENV replication, we generated specific RRM deletion mutants of PACT. PACT RRM mutants lacking either RRM1 or RRM2/3 failed to rescue DENV replication to the same extent as the wildtype protein, indicating that all three motifs are possibly important for DENV replication (Figure 4D,E). Collectively, these findings demonstrate a pro-viral role for PACT in DENV infection.

### 3.5. PACT Modulates Expression of ER-Associated Pro-Viral Genes

**Figure 5:**
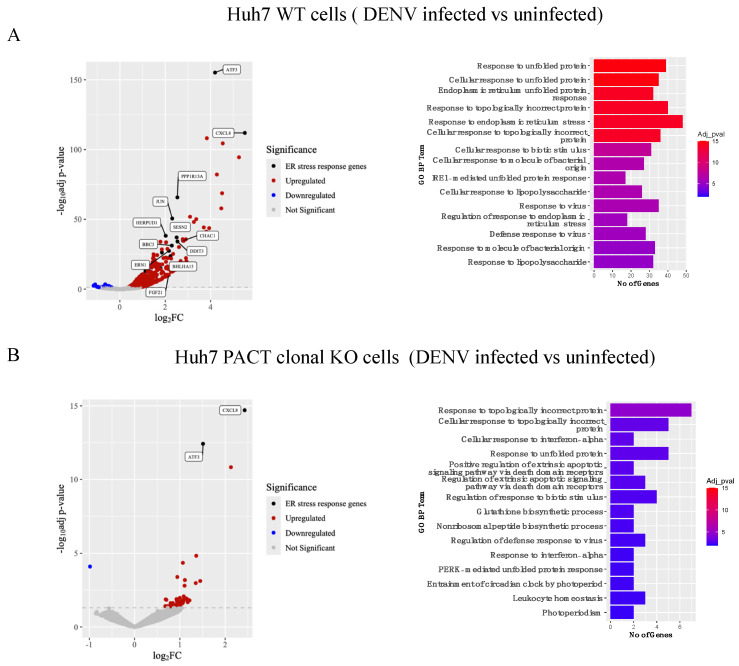
Gene expression data. Differentially expressed genes in uninfected and DENV1 (MOI 0.1) infected (**A**) Huh7 wildtype or (**B**) PACT clonal knockout cells at 48 h post-infection were visualized using volcano plots. ER stress response genes that were more than 2-fold upregulated are labeled. GO enriched genes for each condition are depicted in bar plots.

To gain insights into the possible mechanisms by which PACT could support DENV infection, we analyzed global gene expression changes in uninfected and DENV-infected wildtype and PACT knockout cells using next-generation sequencing (NGS). The results indicated significant upregulation of genes involved in unfolded protein response (UPR) and other ER stress response pathways in wildtype Huh7 cells upon DENV infection, whereas such upregulation was not observed in PACT knockout cells (Figure 5A,B). Notably, several ER-associated pro-viral genes such as Endoplasmic Reticulum to Nucleus Signaling 1 (ERN1/IRE1), DNA Damage Inducible Transcript 3 (DDIT3), Homocysteine Inducible ER Protein With Ubiquitin Like Domain 1 (HERPUD1), and Eukaryotic Translation Initiation Factor 2 Alpha Kinase 3 (EIF2AK3/PERK), which are known to be essential for DENV replication, were upregulated in wildtype cells but not in PACT knockout cells. Further experiments are needed to dissect the role of PACT in modulating the expression of ER-associated pro-viral genes and to elucidate its specific role in DENV infection.

## 4. Discussion

PACT is a double-stranded, RNA-binding protein that plays a multifaceted role in viral infection through modulating host antiviral responses, interacting with viral proteins, and influencing viral replication [19]. PACT activates several anti-viral signaling pathways in the cell in response to infection, including the PKR, RIG-I, and IFN pathways [20]. Viral proteins such as the Ebola VP35, Herpes Simplex Us11, and influenza A NS1 proteins interact with and sequester PACT, thus allowing the virus to evade host immune responses [21,22,23]. Besides activating immune response pathways, PACT can localize viral replication complexes and directly inhibit viral RNA polymerase activity to block viral replication [21]. In a novel strategy, PACT was recently shown to inhibit SARS-CoV-2 replication by blocking cellular GSK3-beta kinase activity and consequently the SARS N protein-Nsp3 interaction, which is critical to viral replication [24].

While PACT plays an antiviral role in all the above instances, our studies demonstrated that PACT exhibits a pro-viral function in the context of DENV infection. Pro-viral effects of PACT have previously been observed in PPRV- and HIV-1-infected cells. PACT promotes PPRV replication through interactions with the viral nucleocapsid protein and subsequent activation of the PKR/eIF2α/SG axis [25]. In the case of HIV-1, PACT interacts with the viral Tat protein and the cellular protein ADAR1 to inhibit PKR activation and promote HIV-1 mRNA translation [26,27,28]. Thus, the effects of PACT on PKR activation and viral replication are variable, depending on the cell type and the virus being used. Previously, PACT has been shown to activate the PKR pathway in Huh7 cells [29], but inhibit PKR activation in HEK293 cells [14]. Since our study demonstrated that PACT promotes DENV replication in both of these cell types, we predict a possible role for PACT in regulating DENV replication through alternative, PKR-independent pathways.

Recent studies have demonstrated that the sequestration of PACT by ZIKV subgenomic RNA fragments (sfRNAs) inhibits the formation of the RISC complex and results in the subsequent degradation of ZIKV RNA [30]. In line with these findings, PACT has also been predicted to interact with DENV sfRNAs in vitro [31,32]. While we did not directly test for interactions of PACT with DENV sfRNAs, we examined if PACT co-localizes with DENV RNA in infected HEK293/FH-PACT-myc-PACT cells by immunostaining. Viral RNA was visualized by staining with the dsRNA-specific J2 antibody, and PACT was visualized by staining with an anti-myc antibody. However, DENV RNA and PACT appeared to localize to distinctly different regions in infected cells (Figure S2). Also, sequestration of PACT by DENV sfRNAs is more likely to have anti-viral effects than the pro-viral effects observed in this study. This led us to explore other genes and pathways that are modulated by PACT in DENV-infected cells using next-generation sequencing.

In our transcriptomics experiments, we observed that ER-associated stress response genes are significantly upregulated in wildtype Huh7 cells in response to DENV infection but not in PACT knockout cells. In fact, mutations in PACT have been shown to dysregulate integrated ER stress response pathways in cells [33]. Since activation of ER stress response pathways, particularly the ERN1/IRE1 and EIF2AK3/PERK pathways, is critical for active flaviviral replication [34,35,36], we predict a possible role for PACT in modulating expression of pro-viral genes involved in these pathways and thereby regulating DENV replication. Future experiments will focus on determining the mechanistic role of PACT in regulating the expression of ER-associated pro-viral factors that are essential for DENV infection.

## Data Availability

The original contributions presented in the study are included in the Supplementary Material, further inquiries can be directed to the corresponding author.

## Supplementary Materials

The following supporting information can be downloaded at https://www.mdpi.com/article/10.3390/v16050725/s1, Table S1: Ranked table of DENV host factors obtained by MAGeCK analysis of sequencing data from the CRISPR screen, along with FDR-corrected *p*-values; Figure S1: Effect of PACT knockout on cell viability. Absorbance at 590 nm is plot for MTT assays performed with WT and PACT knockout Huh7 cells at the indicated time points; Figure S2: Interaction of PACT with viral RNA. Immunostaining of HEK293/FH-PACT/myc-PACT cells infected with DENV2-16681 at a MOI of 1 using J2 and anti-myc antibodies.
