# Supplementary material for: CRISPR Screen Reveals PACT as a Pro-Viral Factor for Dengue Viral Replication
Source: Viruses. 2024 May 3;16(5):725. doi: 10.3390/v16050725 (PMC11125577; doi:10.3390/v16050725)
Supplement: Supplementary file 1 [file viruses-16-00725-s001.zip › viruses-2938353 supplementary figures.pdf]

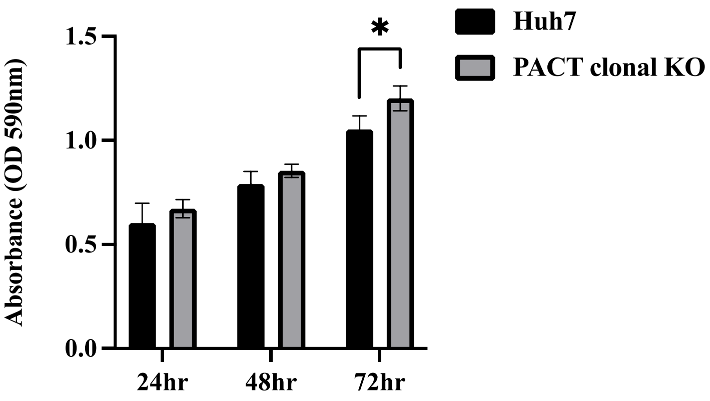

Figure S1. Effect of PACT knockout on cell viability. Absorbance at 590nm is plot for MTT assays performed with WT and PACT knockout Huh7 cells at the indicated time points.

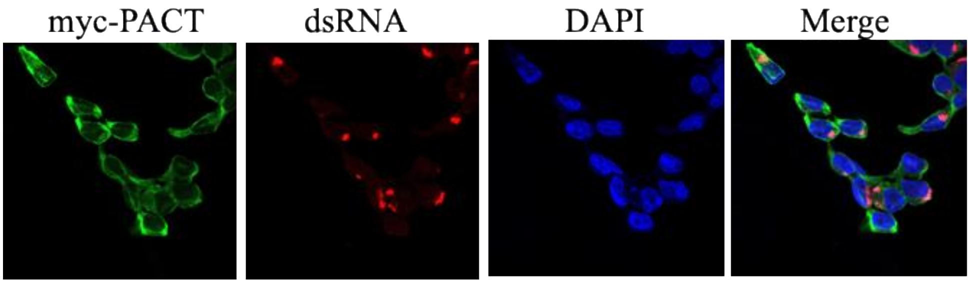

Figure S2. Interaction of PACT with viral RNA. Immunostaining of HEK293/FH-PACT/myc-PACT cells infected with DENV2-16681 at a MOI of 1 using J2 and anti-myc antibodies.
